# Supplementary material for: Fruit-In-Sight: A deep learning-based framework for secondary metabolite class prediction using fruit and leaf images
Source: PLoS One. 2024 Aug 8;19(8):e0308708. doi: 10.1371/journal.pone.0308708 (PMC11309380; doi:10.1371/journal.pone.0308708)
Supplement: Attachment — Submitted filename: response to reviewers_25may2024.docx [file pone.0308708.s002.docx]

**Point-to-point rebuttal**

**Academic Editor:**

1. Please ensure that your manuscript meets PLOS ONE's style requirements, including those for file naming. The PLOS ONE style templates can be found at

<https://journals.plos.org/plosone/s/file?id=wjVg/PLOSOne_formatting_sample_main_body.pdf> and <https://journals.plos.org/plosone/s/file?id=ba62/PLOSOne_formatting_sample_title_authors_affiliations.pdf>

*Response:* We have ensured that the revised manuscript meets the PLOS ONE’s style requirements.

2. Please note that PLOS ONE has specific guidelines on code sharing for submissions in which author-generated code underpins the findings in the manuscript. In these cases, all author-generated code must be made available without restrictions upon publication of the work. Please review our guidelines at [https://journals.plos.org/plosone/s/materials-and-software-sharing#loc-sharing-code](https://journals.plos.org/plosone/s/materials-and-software-sharing#_blank) and ensure that your code is shared in a way that follows best practice and facilitates reproducibility and reuse.

*Response:* We have deposited the codes in GitHub (https://github.com/binaypanda/Fruit-In-Sight), and added the same under the Data Availability section of the submission.

3. Thank you for stating the following financial disclosure:

"Research reported in this manuscript is funded by an extramural grant from the Department of Biotechnology, Government of India to BP (BT/PR36744/BID/7/944/2020)."

Please state what role the funders took in the study.  If the funders had no role, please state: "The funders had no role in study design, data collection and analysis, decision to publish, or preparation of the manuscript."

If this statement is not correct you must amend it as needed.

Please include this amended Role of Funder statement in your cover letter; we will change the online submission form on your behalf.

*Response*: We have added the following disclosure - The funders had no role in study design, data collection and analysis, decision to publish, or preparation of the manuscript.

4. When completing the data availability statement of the submission form, you indicated that you will make your data available on acceptance. We strongly recommend all authors decide on a data sharing plan before acceptance, as the process can be lengthy and hold up publication timelines. Please note that, though access restrictions are acceptable now, your entire data will need to be made freely accessible if your manuscript is accepted for publication. This policy applies to all data except where public deposition would breach compliance with the protocol approved by your research ethics board. If you are unable to adhere to our open data policy, please kindly revise your statement to explain your reasoning and we will seek the editor's input on an exemption. Please be assured that, once you have provided your new statement, the assessment of your exemption will not hold up the peer review process.

*Response*: All data are publicly available at <https://www.kaggle.com/datasets/binaypandalabmember/plos-one-data/>. We have accordingly modified the Data Availability Statement in the submission form.

5. Please include captions for your Supporting Information files at the end of your manuscript, and update any in-text citations to match accordingly. Please see our Supporting Information guidelines for more information: <http://journals.plos.org/plosone/s/supporting-information>.

*Response*: We have included captions to the Supporting Information files at the end of the manuscript and made changes to the file name and in-text citation as per the journal guidelines

**Reviewers 1 and 2:**

1. Is the manuscript technically sound, and do the data support the conclusions?
The manuscript must describe a technically sound piece of scientific research with data that supports the conclusions. Experiments must have been conducted rigorously, with appropriate controls, replication, and sample sizes. The conclusions must be drawn appropriately based on the data presented.

Reviewer #1: No

Reviewer #2: Yes

*Response*: We conducted the analyses rigorously and with appropriate controls. The sample sizes are indicated in Table 1 and the controls in Table 2. With the same seed, we ensure that the runs are reproducible. Furthermore, we have provided all the codes used in the study on Github (https://github.com/binaypanda/Fruit-In-Sight).

2. Has the statistical analysis been performed appropriately and rigorously?

Reviewer #1: Yes

Reviewer #2: No

*Response*: To ensure the predicted model's rigour, we performed bootstrapping using 10-fold cross-validation for both the single-analyte and multi-analyte frameworks in addition to the original run to avoid any training bias. We report these cross-validation errors (line # 247-248).

3. Have the authors made all data underlying the findings in their manuscript fully available?
The [PLOS Data policy](http://www.plosone.org/static/policies.action#_blank) requires authors to make all data underlying the findings described in their manuscript fully available without restriction, with rare exception (please refer to the Data Availability Statement in the manuscript PDF file). The data should be provided as part of the manuscript or its supporting information, or deposited to a public repository. For example, in addition to summary statistics, the data points behind means, medians and variance measures should be available. If there are restrictions on publicly sharing data—e.g. participant privacy or use of data from a third party—those must be specified.

Reviewer #1: Yes

Reviewer #2: Yes

*Response:* Thank you.

4. Is the manuscript presented in an intelligible fashion and written in standard English?
PLOS ONE does not copyedit accepted manuscripts, so the language in submitted articles must be clear, correct, and unambiguous. Any typographical or grammatical errors should be corrected at revision, so please note any specific errors here.

Reviewer #1: No

Reviewer #2: Yes

*Response*: We have gone over the manuscript carefully and removed typographical and grammatical errors in the revised manuscript.

**Reviewer 1:**

*Comment:* The presented work has limited novelty as already existing approaches have been applied. It is my advice to introduce innovation and novelty in the approach.

*Response*: We appreciate the reviewer’s comments. As a biology lab, we leverage and optimise existing methods for novel applications. Applications of existing algorithms and tools are often crucial in areas where they were not originally intended for. The current study, we believe, is a testament to that approach. One of the most challenging aspects of our research was collecting and preparing a well-annotated dataset for deep-learning applications in agriculture. We dedicated a significant amount of time and effort to cover a vast geographical area (0.6 million sq. km), collecting fruits and leaves, imaging them, and annotating them before using them for the analytical metabolite concentration procedure in the laboratory. As the reviewers will appreciate, this was not without challenges, often in some places with summer temperatures (the tree only fruits during the summer months) of close to 50 degrees C.

The other aspect of our work is a thorough comparison with multiple available tools. Other than the default *YOLOv5m* architecture, we tested five variants: *v0* (the YOLO architecture adapted to detect small objects (http://cs230.stanford.edu/projects_fall_2021/reports/103120671.pdf)), *v1* (modified head by tweaking *v0* and increasing the number of C3 layers in the p2 block from 1 to 3, in the p3 block from 3 to 5, in the p4 block from 3 to 5 and added a p5 block), *v2* (added an extra p2 block to head), *v3* (added 1 extra p2 and p5 blocks each to the head), and *v4* (deleted p5). Using default parameters, we also tested six state-of-the-art architectures under image classification frameworks such as *GoogLeNet*, *Inception* *v3*, *EfficientNet_B0*, *Resnext_50*, *Resnet18* and *SqueezeNet*. The image classification frameworks used cropped images after bounding box detection using the best model obtained from *YOLOv5*.

For the best model, we find compelling evidence that boosting a single-analyte model predicting azadirachtin, with predictions from nine other image-analyte models, significantly increases the azadirachtin prediction sensitivity and results in complete specificity. To our knowledge, this combinatorial approach is a novel method to enhance the prediction accuracy of a model while utilizing multi-dimensional outputs from the same specimen using respective models. Furthermore, we have developed a mobile application that leverages this power to predict neem azadirachtin class in real-time on the field.

The above efforts add innovation and novelty towards a new application to the prevailing object detection and classification paradigms.

*Comment:* Experimental work seems satisfactory

*Response*: Thank you.

**Reviewer 2**:

Authors have identified the best model out of YOLOv5, GoogLeNet,23 InceptionNet, EfficientNet_B0, Resnext_50, Resnet18, and SqueezeNet for detecting metabolite class from fruits and leaf images for plucking in right time. The following points need to be addressed for better understanding by the readers.

*Comment:* The organization need to be improved.

*Response*: We thank the reviewer for this comment. We have gone over the manuscript, organized it better and improved the flow and clarity. All the modified texts are highlighted in the revised submission.

*Comment:* In many location the typo errors are noticed and needs proper care. Some cases the lines are not complete.

*Response*: Thank you. We have gone over the manuscript and removed the typographical and grammar errors.

*Comment:* It’s difficult to identify the contributions made in this manuscript. No conclusion section was available.

*Response*: We thank the reviewer for pointing this out. Following the suggestion, we have added a separate Conclusion section (line # 368-375) and highlighted the findings and contributions made in the manuscript.

*Comment:* Discussion need to be improved.

*Response*: We have re-organized the Discussion section, added additional content (line # 304-319 and line # 346-352) and improved the flow and clarity. The revised portions are highlighted in the text.

*Comment:* Reference are not presented as per the journal standard. Uniformity in author's name, jnl name, page number, year, vol, and page numbers are missing.

*Response*: We have formatted the references as per the journal standard.

*Comment:* Figures are of not good quality.

*Response*: We have uploaded higher quality images.
