# Supplementary material for: Fruit-In-Sight: A deep learning-based framework for secondary metabolite class prediction using fruit and leaf images
Source: PLoS One. 2024 Aug 8;19(8):e0308708. doi: 10.1371/journal.pone.0308708 (PMC11309380; doi:10.1371/journal.pone.0308708)
Supplement: Attachment — Submitted filename: response to reviewers_18jul2024.docx [file pone.0308708.s003.docx]

**Point-to-point rebuttal**

Reviewer's Responses to Questions

**Comments to the Author**

1. If the authors have adequately addressed your comments raised in a previous round of review and you feel that this manuscript is now acceptable for publication, you may indicate that here to bypass the “Comments to the Author” section, enter your conflict of interest statement in the “Confidential to Editor” section, and submit your "Accept" recommendation.

Reviewer #1: (No Response)

2. Is the manuscript technically sound, and do the data support the conclusions?

The manuscript must describe a technically sound piece of scientific research with data that supports the conclusions. Experiments must have been conducted rigorously, with appropriate controls, replication, and sample sizes. The conclusions must be drawn appropriately based on the data presented.

Reviewer #1: Partly

Authors’ response: We conducted the analyses rigorously with appropriate controls and with statistical rigor. Additionally, we have described the methods in detail, made all the data and code openly available in Kaggle (<https://www.kaggle.com/datasets/binaypandalabmember/plos-one-data/> ) and Github ((<https://github.com/binaypanda/Fruit-In-Sight>), and have given all the background information for anyone to reproduce our results. Therefore, we believe that our manuscript presents a technically sound piece of scientific research.

3. Has the statistical analysis been performed appropriately and rigorously?

Reviewer #1: I Don't Know

Authors’ response: As mentioned above, we have conducted the analyses rigorously and with appropriate controls and with statistical rigor, where required.

4. Have the authors made all data underlying the findings in their manuscript fully available?

The [PLOS Data policy](http://www.plosone.org/static/policies.action#sharing) requires authors to make all data underlying the findings described in their manuscript fully available without restriction, with rare exception (please refer to the Data Availability Statement in the manuscript PDF file). The data should be provided as part of the manuscript or its supporting information, or deposited to a public repository. For example, in addition to summary statistics, the data points behind means, medians and variance measures should be available. If there are restrictions on publicly sharing data—e.g. participant privacy or use of data from a third party—those must be specified.

Reviewer #1: Yes

5. Is the manuscript presented in an intelligible fashion and written in standard English?

PLOS ONE does not copyedit accepted manuscripts, so the language in submitted articles must be clear, correct, and unambiguous. Any typographical or grammatical errors should be corrected at revision, so please note any specific errors here.

Reviewer #1: Yes

6. Review Comments to the Author

Please use the space provided to explain your answers to the questions above. You may also include additional comments for the author, including concerns about dual publication, research ethics, or publication ethics. (Please upload your review as an attachment if it exceeds 20,000 characters)

Reviewer #1: Still I am not convinced with the experimentation process and regarding novelty and scientific contribution.
Please look into it and resolve the issue.

Authors’ response: As scientists, we regularly review manuscripts from many journals written by other scientists, and therefore, we respect the reviewer's comments. However, we disagree with the reviewer's comment on "novelty and scientific contribution". As mentioned before, innovations in biology do not only sometimes happen due to the development of new algorithms but also because of how ingenious we are in using the tools to develop new applications. Additionally, the collection of new, good-quality and well-annotated data is something that we must emphasize. The quality of science is often dependent on this. Our manuscript describes a new application of deep-learning tools using a dataset that took years to collect, annotate and analyze. Therefore, when we hear that the reviewer is not convinced, we have to know the details of the points the reviewer is not convinced of.

The revised manuscript and our response to the reviewer's comments earlier on the same point adequately addressed this point. For clarity, we would like to repeat that again below -

*As a biology lab, we leverage and optimise existing methods for novel applications. Applications of existing algorithms and tools are often crucial in areas where they were not originally intended for. The current study, we believe, is a testament to that approach. One of the most challenging aspects of our research was collecting and preparing a well-annotated dataset for deep-learning applications in agriculture. We dedicated a significant amount of time and effort to cover a vast geographical area (0.6 million sq. km), collecting fruits and leaves, imaging them, and annotating them before using them for the analytical metabolite concentration procedure in the laboratory. As the reviewers will appreciate, this was not without challenges, often in some places with summer temperatures (the tree only fruits during the summer months) of close to 50 degrees C.*

*The other aspect of our work is a thorough comparison with multiple available tools. Other than the default YOLOv5m architecture, we tested five variants: v0 (the YOLO architecture adapted to detect small objects (http://cs230.stanford.edu/projects_fall_2021/reports/103120671.pdf)), v1 (modified head by tweaking v0 and increasing the number of C3 layers in the p2 block from 1 to 3, in the p3 block from 3 to 5, in the p4 block from 3 to 5 and added a p5 block), v2 (added an extra p2 block to head), v3 (added 1 extra p2 and p5 blocks each to the head), and v4 (deleted p5). Using default parameters, we also tested six state-of-the-art architectures under image classification frameworks such as GoogLeNet, Inception v3, EfficientNet_B0, Resnext_50, Resnet18 and SqueezeNet. The image classification frameworks used cropped images after bounding box detection using the best model obtained from YOLOv5.*

*For the best model, we find compelling evidence that boosting a single-analyte model predicting azadirachtin, with predictions from nine other image-analyte models, significantly increases the azadirachtin prediction sensitivity and results in complete specificity. To our knowledge, this combinatorial approach is a novel method to enhance the prediction accuracy of a model while utilizing multi-dimensional outputs from the same specimen using respective models. Furthermore, we have developed a mobile application that leverages this power to predict neem azadirachtin class in real-time on the field.*

*The above efforts add innovation and novelty towards a new application to the prevailing object detection and classification paradigms.*

7. PLOS authors have the option to publish the peer review history of their article ([what does this mean?](https://journals.plos.org/plosone/s/editorial-and-peer-review-process#loc-peer-review-history)). If published, this will include your full peer review and any attached files.
If you choose “no”, your identity will remain anonymous but your review may still be made public.


**Do you want your identity to be public for this peer review?** For information about this choice, including consent withdrawal, please see our [Privacy Policy](https://www.plos.org/privacy-policy).

Reviewer #1: No
